# Quality of intrapartum care assessed by women participating in a midwifery model of continuity of care

**DOI:** 10.18332/ejm/134502

**Published:** 2021-04-23

**Authors:** Ingegerd Hildingsson, Annika Karlström, Christine Rubertsson, Birgitta Larsson

**Affiliations:** 1 Department of Women’s and Children’s Health, Uppsala University, Uppsala, Sweden; 2 Department of Nursing, Mid Sweden University, Sundsvall, Sweden; 3 Department of Health Science, Faculty of Medicine, Lund University, Lund, Sweden; 4 Sophiahemmet University College, Stockholm, Sweden

**Keywords:** continuity, intrapartum care, midwifery, rural, quality of care

## Abstract

**INTRODUCTION:**

Continuity models are rare in Sweden. The aim was to compare the intrapartum care experiences between women who had or not a known midwife attending their birth.

**METHODS:**

A cohort study was conducted in a rural area with long distance to a labor ward in Sweden. From August 2017 to June 2019, a continuity model with a known midwife was offered between 7 a.m. and 11 p.m. daily. Questions about intrapartum care were assessed in two aspects; the perceived reality and the subjective importance.

**RESULTS:**

A total of 226 women recruited in early pregnancy were followed up two months after giving birth. Women who had a known midwife providing labor care reported higher overall satisfaction and were more likely to value the subjective importance and the perceived reality significantly higher than women who received intrapartum care without a known midwife assisting. When analyzing the medical aspects of intrapartum care, the most important factors for not being satisfied were deficiencies in the partner’s involvement and insufficient pain relief. For the emotional aspects, deficiencies in participation in decision making was the most important aspect.

**CONCLUSIONS:**

Having a known midwife assisting at birth reduced discrepancies between women’s subjective importance and perceived reality of intrapartum care, especially regarding support and the involvement of the partner. A known midwife generated higher overall satisfaction with the medical and emotional aspects of intrapartum care. To improve satisfaction and the quality of intrapartum care, continuity midwifery models of care should be implemented.

## INTRODUCTION

Intrapartum care should be designed with a minimum of interventions so as to result in a healthy mother and baby and a positive birth experience^1^. Previous research has shown several factors associated with a positive experience of birth and intrapartum care, such as having one’s expectations fulfilled^2,3^ and having a normal birth without interventions^4^. Some aspects seem to be of the greatest importance, such as the availability of support during labor and birth. Continuous support is not only convenient for the woman, it also results in less need for pain relief, shorter length of labor, more normal births and a better outcome for the baby, according to a Cochrane Review^5^. Women value continuous support from the accompanying person as well as from health professionals^6^. Still, the content of the support and characteristics of the support persons are also shown to be important^6,7^. While partners are important to support persons, their needs and preferences are not always acknowledged. An integrative literature review of 25 studies demonstrated that partners want to be informed, involved and respected and call for family-oriented care^8^.

Continuity models of midwifery care are another important aspect that could result in greater satisfaction, fewer interventions, more spontaneous births, less need for epidural and more cost-effective care, as shown in a Cochrane Review of 15 high-quality studies comprising more than 17000 women^9^. Perriman et al.^10^ emphasized the woman–midwife relationship as the most important aspect in continuity models. It entails individualized care based on a trustful relationship, which is empowering for women.

Labor pain could be managed in several ways. Women’s experiences of pain relief methods showed that pharmacological methods might reduce the pain but could result in negative side effects. On the other hand, non-pharmacological methods could increase bonding with staff and support persons^11^. Managing labor pain could be influenced by individualized continuous support and acceptance^12^. Feelings of control and being involved in decision making are other important aspects of intrapartum care, as shown in a large systematic review comprising 35 studies^6^.

During pregnancy and childbirth, women and their partners are exposed to healthcare, mainly without being ‘sick’. Healthcare for pregnancy and birth, on the other hand, is organized within healthcare facilities designed for patients. Usually, becoming parents are active participants, and antenatal and intrapartum care provide an excellent venue for developing a person (or women/family)-centered approach to improve the quality of care. Person-centered care is a concept adopted in the majority of health organizations in Sweden. It requires that the individual is seen, heard and involved in the care, based on their needs, resources and prerequisites^13^. There has also been a call for developing models with continuity^14^, and this study reports on one such initiative.

### Care context

The Swedish healthcare system is organized within two sectors, primary healthcare in outpatient clinics in the community, and specialist care in hospitals. Midwives usually work in either of these healthcare systems and seldom rotate between the systems. Midwives are the primary caregivers during antenatal care, and women follow a basic schedule consisting of 8 to 9 visits. In normal pregnancies, there are no visits to a doctor. If a woman develops complications during pregnancy or have previous healthcare problems affecting the pregnancy, the midwife refers the woman for consultation, usually with an obstetrician. After the consultation, the antenatal midwife works in collaboration with the obstetrician, if needed. Women usually have satisfactory caregiver continuity during pregnancy, but there is no continuity of caregiver between antenatal and intrapartum care. Midwives are also the main caregivers for normal births in hospitals. They work in a highly medicalized intrapartum setting, taking care of several women, limiting the opportunities to be present in the labor room and supporting them. In case of complicated pregnancies and births, midwives collaborate with obstetricians but manage the birth. Obstetricians perform caesarean sections, and the midwife is present and takes care of the baby and the family after surgery. The Swedish midwife could perform instrumental vaginal births (vacuum extractions), but an obstetrician in most hospitals manages these. There are no alternative birth settings in Sweden, and the number of homebirths is very low (less in 1/1000). In the last 20 years, nearly all smaller labor wards in Sweden have been closed, resulting in long travel distances to hospitals with labor wards^15^.

Midwifery care during pregnancy and birth is rather fragmented in the Swedish system. Given the lack of coherent midwifery models, this study aimed to compare the intrapartum care experiences between women who had or not a known midwife attending their birth.

An additional aim was to identify deficiencies in intrapartum care quality concerning women’s background and what aspects mattered the most when they were not very satisfied with intrapartum care.

## METHODS

### Design

This is a cohort study where pregnant women were recruited in early pregnancy and followed up after birth. The women were offered to have a midwifery care model from a known midwife throughout antenatal and intrapartum care. The opportunity to have a known midwife at labor and birth was limited to certain hours each day.

### Setting

The continuity of care project started in February 2017, shortly after closing one of the labor wards in the area. The antenatal clinic remained in the hospital, together with an outpatient gynecological ward. The small city where the study took place is located in a rural area in northern Sweden. Before the closure, the labor ward had around 350 births a year. The consequence of closing was that women had to travel 100–120 km to give birth in the two remaining labor wards in the region. Four midwives (three who worked 75% and one who worked 100%), provided antenatal care. The antenatal clinic was open 8 a.m. to 5 p.m. during weekdays. From 1 August 2017 until 30 June 2019, the midwives were on-call for births every day on a rotation schedule, which meant that one of the midwives travelled to the hospital of the woman’s choice to provide care during the labor and birth. One midwife was on call each day between 7 a.m. and 11 p.m. but due to lack of staff, the on-call service was not available during the night-time hours. The service was also restricted during two summer months, where only two midwives were working. After being on-call for 12 hours, the midwife had to hand over the birth to the hospital staff due to Swedish worktime regulations. If there was no ongoing birth, the midwife on-call was available on the telephone or at the antenatal clinic if a woman needed a check-up that did not require immediate hospital admission (e.g. water discharge without contractions or reduced fetal movements). In such cases, the midwife could communicate with the hospital staff through electronic records and telephone.

### Recruitment of participants

Women were informed about the project when they contacted the antenatal clinic for a booking appointment. There was also information available on web pages and folders in the clinic’s waiting room. Women who consented to participate were assigned a primary midwife, whom they met during the six to nine antenatal visits recommended in the national guidelines^16^. They also met the other midwives involved in the project when attending information meetings, antenatal classes or at the clinic during check-ups. It was necessary to communicate in Swedish by telephone to be part of the project, leading to the exclusion of asylum seekers and foreign-born women who were not eligible for participation.

Women identified with fear of birth were prioritized in the project, as a known midwife could be extremely important for these women^17^. Another prioritization was women expecting the first baby, as it is known that continuity with a known midwife could improve birth outcome in first-time mothers^18^.

At the onset of labor or consultation during the latent phase, the woman contacted the midwife on-call, who had a telephone with excellent coverage, similar to those used by paramedics in the mountains. This special communication equipment was important, as, along with some parts of the roads leading to the hospitals, there was no mobile phone coverage. The midwife on-call travelled in a specially designed car with equipment for emergency births, and the parents travelled in their car. When labor started during the night, the couple went to the hospital themselves. In such cases, the hospital staff usually called the midwife on call and informed about the woman.

### Data collection

Data were collected by two questionnaires, the first in mid-pregnancy after the routine ultrasound examination and the second two months after the birth. The questionnaires were sent to each woman’s home address with a prepaid-response envelope. Two reminders were later sent by text messages. In the first questionnaire, background data were collected (age, marital status, country of birth, education level, and parity). Women assessed their physical and emotional health on a 4-point Likert scale with response options: ‘1=Very good’, ‘2=Good’, ‘3=Bad’, and ‘4=Very bad’. After that, the answers were dichotomized into ‘Good’ (1+2) and ‘Not good’ (3+4).

Women’s emotional wellbeing was investigated through the Edinburgh Postnatal Depression Scale (EPDS). The EPDS includes ten questions to identify women with symptoms of depression and anxiety, and a cut-off point of 13 or more was used as recommended when used during pregnancy^19^. Fear of birth was assessed using the Fear of Birth Scale (FOBS), and a cut-off point of 60 or more was used to classify women with fear of birth^20-21^. Two months after the birth of the baby, each woman received a second questionnaire that evaluated the experience of labor and birth, (onset of labor, mode of birth, length of labor, self-reported birth complications, use of epidural anesthesia and whether the woman had a known midwife providing her labor care).

To investigate the women’s rating of the quality of intrapartum care, they were told to evaluate several statements about the content of intrapartum care (information, support, midwife being present, the involvement of the partner in the care, pain relief, participation in decision making, perception of control, breastfeeding support, and to talk through the birth afterwards). These ratings were assessed in two ways, using the intrapartum version of the validated instrument Quality from the Patient’s Perspective (QPP)^22^.

First, the perceived reality (PR) mirrored women’s experiences regarding the actual care received. Second, they assessed the subjective importance (SI) of the same statement, e.g. how important the particular aspect was to them. Evaluating statements in two ways like this has previously been utilized in studies about intrapartum care^23,24^. The perceived reality (PR) had four response categories ranging from ‘Do not agree at all’ to ‘Agree’. The response options for the subjective importance ranged from ‘Of no or minor importance’ to ‘Of very great importance’. After that, an index was created by combing the answers from SI and PR for each question that yield seven levels of assessed quality for each statement. Based on the instructions from the creators of the Quality of Care from the Patients’ Perspective (QPP)^25^, the index was divided into three categories: 1) ‘Deficient quality of care’ indicates aspects of care the respondent assessed as important but lacking in the care received, 2) ‘Balanced quality of care’ was received when the perceived reality and the subjective importance were in line, and 3) ‘Excessive quality of care’ occurred when the given care was beyond the expectations of importance (e.g. better than expected). The original QPP creators recommend that if more than 20% report ‘Deficient quality of care’, action should be taken to improve these aspects^25^. The three-part quality index was dichotomized into ‘Deficient care’ versus ‘Not deficient care’.

Moreover, the questionnaire covered some overall aspects of the medical and emotional aspects of intrapartum care. The Swedish midwifery model involves independent work within the two health systems. Thus, midwives are responsible for the medical and emotional aspects of care and work in collaboration with other health professions such as obstetricians or anesthesiologists. Examples of the medical aspects were check-ups of labor progress, pain relief, and monitoring the baby. Examples of the emotional aspects were support, encouragement, and involvement in decision making. These questions were reported on a 5-point rating scale ranging from ‘Very satisfied’ to ‘Very dissatisfied’. The questions were dichotomized into ‘Very satisfied’ and ‘Less than very satisfied’ for the analysis. The dichotomization basis came from previous studies suggesting that answers less than ‘Very satisfied’ could indicate areas of improvement^26^. The questions’ reliability was assessed with the Cronbach’s alpha coefficient, which was 0.80 for PR and 0.75 for SI.

### Data analysis

Descriptive statistics were used to present the background data of the participants. Paired sample t-tests were calculated for the differences in mean scores between PR and SI for each statement. Thereafter, independent sample t-tests were used to detect differences in PR and SI between women who had a known midwife during labor and birth and those who did not. Chi-squared tests or Fisher’s exact tests were then used to compare proportions of the three levels of quality of intrapartum care (deficient, balanced and excessive-quality). Finally, in order to find statements most strongly associated with dissatisfaction with the medical and emotional aspects of intrapartum care in relation to having a known midwife or not, logistic regression analysis was used. The statistical analyses were conducted using the Statistical Package for Social Sciences version 25. The study was approved by the Regional Ethics Board, DNR 2017 / 120-31.

## RESULTS

Initially, 314 women consented to participate in the project. The follow-up questionnaire was sent to 278 women after excluding women with miscarriage (n=23) and women who withdrew or moved from the area (n=13). The response rate to the follow-up questionnaire was 85% (n=236/278). Analysis of non-responders showed that women who did not return the follow-up questionnaire (n=42) were more likely to be born outside Sweden (p=0.000) and had not completed the first questionnaire (p=0.000). No other background differences were found. Seventy-seven women (34%) responded that they had a known midwife during labor and birth (the primary midwife or one of the other project midwives they met during pregnancy). Also, ten of the 236 questionnaires were excluded from further analysis. The reasons for exclusion were that four women gave birth before the on-call service started, four women gave birth outside the county council, and two did not complete the intrapartum care questions.

Table 1 shows the background characteristics of the women in the project. The majority were aged 25–35 years, living with a partner and born in Sweden. The most common level of education was high school, and there were more multiparous women than primiparous. When self-rating health, few women rated their physical health as being less than good (6%), and the proportion of women who rated their emotional health as not good was twice as high (12%), a finding confirmed in the proportion of women (12%) with an EPDS-score of 13 or more. In addition, more than one in three women was identified with fear of birth.

**Table 1:** Background of the participants (N=226)

*Characteristics*	*n (%)*
**Age** (years)	
25	35 (15.5)
25–35	156 (69.0)
35	35 (15.5)
**Civil status**	
Living with a partner	214 (94.7)
Not living with a partner	12 (5.3)
**Country of birth**	
Sweden	211 (93.4)
Other	15 (6.6)
**Education level**	
High school or lower	140 (62.5)
University education	84 (37.5)
**Parity**	
Primiparas	93 (41.2)
Multiparas	133 (58.8)
**Self-rated physical health**	
Good	205 (93.6)
Not good	14 (6.4)
**Self-rated mental health**	
Good	192 (87.7)
Not good	27 (12.3)
**Fear of birth**	
FOBS <60	148 (67.3)
FOBS ≥60	72 (32.7)
**EPDS**	
0–12	192 (87.7)
≥13	27 (12.3)
**Onset of labour**^a^	
Spontaneous	144 (63.7)
Induction	72 (31.9)
**Mode of birth**	
Vaginal	179 (79.2)
Instrumental vaginal	13 (5.8)
Elective caesarean section	13 (5.8)
Emergency caesarean section	21 (9.3)
**Length of labour** (hours)	
≤12	179 (84.8)
≥12	32 (15.2)
**Epidural anaesthesia**	
Yes	78 (35.1)
No	144 (64.9)
**Self-reported birth complication**	
Yes	83 (38.6)
No	132 (61.4)

*Number might not add up to 100% due to internal missing values.

a Caesarean sections excluded.

The majority of women had a spontaneous onset of labor (67%), and 79% had a normal vaginal birth. The proportion of caesarean section was 15% in total, with the majority (15%) as an emergency caesarean section performed during labor. Similar proportions of women (5.8%) had an elective caesarean section or instrumental vaginal birth. More than one-third (35%) used epidural for pain relief, and 39% self-reported a birth complication, which comprised anything from minor sutured perineal tears to a prolapsed cord.

### Differences between subjective importance (SI) and perceived reality (PR)

First, each aspect of intrapartum care was explored regarding women’s ratings of the subjective importance compared to the perceived reality using a paired sample t-test between SI and PR, which showed discrepancies in some of the variables. In general, women rated the subjective importance (SI) higher than the perceived reality (PR), e.g. they scored higher on the importance of the aspects under study than their perception of the care received.

The largest discrepancies between women’s ratings were found in the perception of control (SI=3.31, PR=2.77, p=0.000), information (SI=3.56, PR=3.41, p=0.017), the possibility to talk about the birth with the assisting midwife afterwards (SI=2.89, PR=2.58, p=0.001) and sufficient help with the initiation and first breastfeeding (SI=3.06, PR=2.73, p=0.000), where the subjective importance showed higher mean scores than the perceived reality.

### Subjective importance and perceived reality of intrapartum care in relation to continuity with a known midwife

In order to further explore the value of continuity, comparisons were made between women with and without a known midwife assisting during labor and birth for the two aspects (SI and PR) of assessment of the content of intrapartum care (Table 2).

**Table 2:** Subjective Importance and Perceived Reality of aspects of intrapartum care in relation to continuity

	*Known midwife*		*No known midwife*		*Difference in SI*	*Difference in PR*
	*SI*	*PR*	*SI*	*PR*	*(if known midwife or not)*	*(if known midwife or not)*
	*Mean (SD)*	*Mean (SD)*	*Mean (SD)*	*Mean (SD)*	*p*	*p*
The midwife I met most of the time gave me the best possible support during labour and birth	3.31 (0.80)	3.74 (0.59)	3.17 (0.89)	3.21 (0.98)	0.286	0.000
I had good opportunity to participate in decision-making about the birth	3.50 (0.76)	3.42 (0.76)	3.19 (0.82)	3.16 (0.93)	0.000	0.036
I felt in control during labor and birth	3.47 (0.67)	3.01 (0.91)	3.21 (0.75)	2.63 (1.08)	0.019	0.005
The midwife was present in the room as much as I wanted during labor and birth	3.59 (0.72)	3.75 (0.61)	3.34 (0.72)	3.31 (0.96)	0.025	0.000
I got sufficient information about the progress of labor	3.59 (0.62)	3.60 (0.79)	3.52 (0.62)	3.30 (0.90)	0.465	0.016
I got the pain relief I wished for during labor and birth	3.57 (0.71)	3.46 (0.73)	3.46 (1.02)	3.38 (1.00)	0.304	0.602
The midwife I met most of the time made my partner feel involved	3.49 (0.80)	3.50 (0.85)	3.44 (0.78)	3.28 (0.93)	0.677	0.109
I had the opportunity to talk through the birth afterwards, with the assisting midwife	3.15 (0.95)	3.08 (1.08)	2.73 (1.03)	2.82 (1.22)	0.005	0.000
I got the best possible help when the baby was breastfeeding the first time after birth	3.31 (0.95)	2.95 (1.09)	2.93 (1.04)	2.61 (1.16)	0.015	0.053

When women with and without a known midwife were compared regarding the subjective importance of the statements about intrapartum care, the results showed statistically significant differences for five of the variables studied (participation in decision making, perception of control, the midwife being present in the room, the possibility to talk through the birth with the midwife afterwards and help with the first breastfeeding), where women who had a known midwife rated these statements higher compared to women not having a known midwife at birth (Table 2).

Women who had a known midwife also scored higher in five of the statements regarding the perceived reality (their experience of the care): support from the midwife, perception of control, midwife was present as much as they wanted, information about the progress of labor, and the possibility to talk about the birth with the midwife afterwards. These differences were statistically significant (Table 2).

### Quality of intrapartum care

The quality index based on the aspects of intrapartum care (SI and PR) showed that most women rated the quality of intrapartum care as balanced (Table 3). The most balanced aspects were the involvement of the partner, the midwife’s presence and information, with 61–70% reporting a balanced quality of care. However, six out of ten variables were reported as deficient (>20%). Most deficient was the perception of control, followed by the possibility to talk through the birth with the assisting midwife afterwards and getting sufficient help with the initiation and first breastfeeding. Some aspects were also rated as excessive-quality, e.g. better than expected, especially the support from the midwife, the possibility to talk through the birth with the assisting midwife afterwards, and the midwife’s presence (23–30% excessive-quality of care). When comparing women with and without a known midwife assisting during labor and birth, women who had a known midwife were less likely to rate the support from the midwife and the involvement of the partner as deficient (Table 3).

**Table 3:** Rank order of women’s perception of intrapartum care based on an index of perceived reality and subjective importance

	*Deficient care*	*Balanced care*	Excessive care*	*Known midwife*	*Not known midwife*	*Reporting deficient care when having a known midwife*
	*Per cent of all women in the study*			*%^a^*	*%^a^*	*OR (95% CI)*
The midwife made my partner involved during labor and birth	18	70	12	9/79/12	23/65/12	0.38 (0.15–0.96)*
The midwife was present as much as I wanted	16	61	23	8/72/20	20/55/25	0.41 (0.16–1.07)
I got sufficient information during labor and birth	22	61	17	15/62/23	26/60/14	0.50 (0.23–1.08)
I got the best possible support from the midwife	13	57	30	3/62/34	20/53/27	0.17 (0.03–0.75)**
I received the pain relief method preferred	22	56	22	22/59/19	23/54/24	1.04 (0.52–2.10)
I was involved in decision making during labor and birth	25	54	21	21/50/19	27/50/23	0.84 (0.42–1.70)
I perceived that I had control over my body during labor and birth	43	52	5	37/61/2	45/47/8	0.75 (0.42–1.34)
I got the best possible support when I breastfed the first time after birth	34	48	18	33/48/19	34/49/17	0.95 (0.51–1.76)
I had the opportunity to talk through the birth with the assisting midwife	37	36	27	30/47/23	41/30/30	0.64 (0.34–1.20)

* Deficient, balanced and excessive care is based on an index combining perceived reality and subjective importance (Quality from the Patient’s Perspective, QPP)^22^.

a Percentage refers to Deficient/Balanced/Excessive quality of care

### Most important aspects of not being very satisfied with intrapartum care

Altogether, 42.5% of the women in the project were dissatisfied (e.g. less than very satisfied) with the medical aspects of care, and 45.8% were dissatisfied with the emotional aspects. In the group of women who had a known midwife, the proportions of being dissatisfied were 31.5% versus 35.1%, respectively. There were statistically significant differences in women who had a known midwife assisting during birth or not, with a p=0.017 for the medical aspects and p=0.023 for the emotional aspects.

Table 4 shows the odds ratios for the two groups with 95% confidence interval for being ‘less than very satisfied’ with the medical and emotional aspects. The factors that contributed most to being ‘less than very satisfied’ with the medical aspects were lack of partner being involved by the midwife and not sufficient pain relief. In addition, in those who did not receive care from a known midwife, dissatisfaction with the presence of a midwife in the room, as much as the woman wanted, and dissatisfaction with participation in decision making also contributed to not being satisfied.

**Table 4:** Women’s dissatisfaction with the medical and emotional aspects of intrapartum care in relation to continuity

	*Medical aspects of intrapartum care*		*Emotional aspects of intrapartum care*	
	*OR (95% CI)*	*OR (95% CI)*	*OR (95% CI)*	*OR (95% CI)*
Not reporting deficient quality of care (Ref.)				
Participation in decision making		5.24 (1.71–1.09)***	4.39 (1.09–17.65)*	11.26 (3.06–41.42)***
Partner being involved by the midwife	18.16 (1.87–179.83)*	10.56 (2.13–52.25)***		7.07 (1.83–27.24)**
Sufficient pain relief	5.82 (1.66–20.43)**	8.63 (2.16–34.46)**		
The presence of the midwife		5.23 (1.41–19.35)*		
Support from the midwife				7.71 (1.54–38.49)**
Information about the progress of labor			6.15 (1.07–35.11)*	

* p<0.05,

** p<0.01,

*** p<0.001.

Ref.: reference.

For the emotional aspects of intrapartum care, the highest odds for not being very satisfied was scoring participation in decision making as deficient. This was found in both groups of women. For women with a known midwife, dissatisfaction with the information about the progress of labor also contributed to being less than very satisfied. Two other variables contributed to dissatisfaction in the group without a known midwife, namely deficiencies in the partner being involved by the midwife and the support from the midwife.

## DISCUSSION

The aim of the present study was to compare the intrapartum care experiences between women who had or not a known midwife attending their birth and to identify deficiencies in the quality of intrapartum care. This result highlights the importance of a known midwife as central for high-quality intrapartum care, especially regarding support from the midwife, as women rated the experience of support higher than what they had expected. The study also identified certain quality aspects that were deficient and what mattered the most in women’s assessment of intrapartum care.

### Differences between subjective importance and perceived reality in intrapartum care

The results showed that women rated the subjective importance (SI) higher than the perceived reality (PR). This agrees with other studies that have used instruments measuring both aspects^23,24,27,28^. Discrepancies between SI and PR have been most prominent when it comes to information, involvement in decision making and perception of control^28^. Although intrapartum care generally produces high satisfaction, there are still areas that need improvement if the goal is to provide women-centered care^14^.

### Differences between subjective importance and perceived reality when having a known midwife or not assisting during labor and birth

The differences shown in the subjective importance between women having a known midwife or not were participating in decision making, perception of control, the midwife being present, talking through the birth afterwards with the midwife and help with the first breastfeeding. Similar findings have been reported in an interview study from Western Australia, where it was shown that women who chose continuity models of care knew what they aimed for regarding their pregnancy and birth experience: a care provider that delivered continuity, a relationship with the midwife, and sharing the same birth philosophy^29^. Another explanation could be that women who received care from a known midwife rated these aspects as more important when asked in retrospect.

The differences between women who had a known midwife and those who did not were also mirrored in the perceived reality, where women who had a known midwife were more likely to report that they received the care they wanted. One notable factor was the midwife’s support, where the actual care received was much better than women had hoped for. Such support is strongly connected to the midwife–woman relationship and has been shown in several studies as being the most important component in intrapartum care^29-32^.

Women who had a known midwife also scored higher in participation in decision making, perception of control, and the midwife’s presence. In addition, sufficient information and the opportunity to talk through the birth with the assisting midwife were other areas that were favored when having a known midwife, compared to women who did not have a known midwife. Similar findings have been reported in a previous Swedish study that showed that women with fear of birth who participated in an experimental study where the counselling midwife also attended the birth were more likely to report higher satisfaction with information, participation in decision making, and feelings of control, if they knew the midwife beforehand^17^. Many of the areas are closely interrelated, such as the perception of control, participation in decision-making, and information.

Some aspects, such as the perception of control, reflect a feeling only the woman herself could judge. It might be impossible to change a person’s thoughts and feelings, but there are ways to provide care that empower women. Previous studies have shown that feelings of being in control are enhanced through sufficient information that is tailored to the individual^28,33^. Another important aspect of feelings of being in control is linked to participation in decision making^10,28,34^. Participation in decision making is a crucial concept in the model of person-centered (or in this case woman-centered) care, a concept adopted by Swedish health authorities, and person-centered care also takes into account a person’s life history, values, expectations and views in a holistic way^13,14^.

The presence of the midwife in the room (as much as the woman wanted) was another aspect where there were differences between women having a known midwife or not. The perceived reality showed that women who had a known midwife also had a higher degree of presence, which is inherent in the continuity model. Midwives in the project only assisted ‘their own’ women, while the ward midwives had to take care of several women. Taking individual care of only one family at a time is highlighted by the Swedish Association of Midwives as one of the most important aspects to develop intrapartum care and to make midwives satisfied and willing to stay or return to the profession^35^. Continuity models clearly could make this happen. In addition, the benefits of continuity on women’s and children’s health are well known^9^.

Some of the aspects might be more difficult to change than others, e.g. women’s feelings, as they are always subjective. Other aspects might be able to change without cost, such as sufficient information, the ability to talk through the birth with the assisting midwife and participating in decision making. A telephone call after birth, in which the woman can ask questions/express her concerns or just get the assurance/evaluation of normality could be important, or even more importantly when a birth has been traumatic to refer for additional consultations, as shown in a study from Australia. The authors of that randomized controlled study found that a follow-up telephone call four to six weeks after the birth decreased trauma, depressive symptoms, stress, and self-blame^36^.

### Quality of intrapartum care

Women who had a known midwife assisting during labor and birth were less likely to assess intrapartum care quality as deficient in two areas. First is the involvement of the partner, and second is the support from the midwife. Studies on continuity models have reported that the midwife becomes more like a special friend by getting to know the woman’s family and network^37,38^; couples reported that the relationship with the midwife was viewed as ‘a professional friendship, characterized by equality and inclusiveness’, circumstances that also made the partner feel involved.

Some of the statements were perceived with both deficient quality and excessive-quality (participation in decision making, pain relief, and talking through the birth). This might be explained by the individual midwives’ approach and ability to involve women. In a regional cohort study of 1049 women whose expectations were investigated in late pregnancy and their experiences after birth^31^, it was found that support from the midwife, participating in decision making, perception of control, and the midwife’s presence, were assessed as both ‘worse than expected’ (e.g. deficient quality) and ‘better than expected’ (e.g. excessive-quality). Some of these aspects could be affected by the workload on the labor ward, and there is always a challenge to provide sufficient care under rough circumstances. However, it is likely that parents will accept that the midwife has a heavy workload as long as they feel well informed and cared for.

### The most important factors for the medical and emotional aspects of intrapartum care

Two variables were assessed as important for both the medical and emotional aspects of intrapartum care, namely participation in decision making and involvement of the partner, regardless of whether there was a known midwife assisting or not. The presence of the midwife and the support from the midwife loaded on each domain of intrapartum care but are two sides of the same coin. These were the main attributes for the group of women who did not have a known midwife, and the results point out the importance of the relationship between the woman and the midwife. Such a relationship is inherent in continuity models and is the key to build confidence, which is linked to feelings of being in control^29,34^. Moving the strong focus from the professionals to the ‘patients’, as recommended in the concept of person-centered (or woman-centered) care, could be challenging for healthcare providers, especially in a medically dominated area such as childbirth, where rules and regulations often do not prioritize women’s expectations and experiences.

### Limitations

This study is compromised by its non-randomized design, the self-reported nature of the questionnaires and the fairly low proportion of women who actually received continuity with a known midwife. In the area where the study was carried out, there were not enough numbers of pregnant women or midwives to perform a randomized controlled trial. The lack of midwives made the project vulnerable in cases of sick leave. This was one explanation for the low percentage of continuity. Another explanation was the limited on-call hours, with only 16 out of 24 hours covered in the on-call schedule. This was partly due to work-time regulations. Midwives working in antenatal care not only serve women during and after the pregnancy, they also see women for family planning, and pap smears and the booked receptions give limited space for replacing sick colleagues on call. The project midwives sometimes also had to cover for midwives in standard care, which impacted their possibility to provide intrapartum care to ‘their women’ during births. The long travel distance to the nearest hospital is another issue that might impact the midwives’ willingness to travel 100–120 km by the end of the day. The Swedish context of antenatal and intrapartum care delivered by midwives independently includes both medical and emotional aspects of care that might not be transferable to other midwifery settings or contexts. Despite these circumstances, the results point in the same direction as the international literature, with higher satisfaction when having a known midwife^9^.

## CONCLUSIONS

This study showed that a midwife providing intrapartum care reduced discrepancies between women’s subjective importance and the perceived reality of intrapartum care. Deficiencies in many aspects of intrapartum care were identified but resulted in less disparity if a known midwife assisted the birth, especially regarding support and involvement of the partner. A known midwife generated higher overall satisfaction with the medical and emotional aspects of intrapartum care. Providing women with options of continuity of care, which is known to be the best evidence-based care, should be implemented to offer women and their partners a safe and satisfying experience of intrapartum care. More research about midwifery continuity models is needed from a Swedish perspective and would be of importance to politicians, managers and policymakers, as it might save money and make midwives stay in the profession.
